# Peripheral Inflammation is Associated with Altered Substantia Nigra Activity and Psychomotor Slowing in Humans

**DOI:** 10.1016/j.biopsych.2007.12.007

**Published:** 2008-06-01

**Authors:** Lena Brydon, Neil A. Harrison, Cicely Walker, Andrew Steptoe, Hugo D. Critchley

**Affiliations:** a Department of Epidemiology and Public Health, Institute of Neurology at University College London, London, United Kingdom; b Institute of Cognitive Neuroscience, Institute of Neurology at University College London, London, United Kingdom; c Wellcome Trust Centre for Neuroimaging, Institute of Neurology at University College London, London, United Kingdom; d Brighton and Sussex Medical School, University of Sussex, Brighton, United Kingdom.

**Keywords:** Cytokines, fMRI, peripheral inflammation, psychomotor slowing, substantia nigra

## Abstract

**Background:**

Systemic infections commonly cause sickness symptoms including psychomotor retardation. Inflammatory cytokines released during the innate immune response are implicated in the communication of peripheral inflammatory signals to the brain.

**Methods:**

We used functional magnetic resonance brain imaging (fMRI) to investigate neural effects of peripheral inflammation following typhoid vaccination in 16 healthy men, using a double-blind, randomized, crossover-controlled design.

**Results:**

Vaccination had no global effect on neurovascular coupling but markedly perturbed neural reactivity within substantia nigra during low-level visual stimulation. During a cognitive task, individuals in whom typhoid vaccination engendered higher levels of circulating interleukin-6 had significantly slower reaction time responses. Prolonged reaction times and larger interleukin-6 responses were associated with evoked neural activity within substantia nigra.

**Conclusions:**

Our findings provide mechanistic insights into the interaction between inflammation and neurocognitive performance, specifically implicating circulating cytokines and midbrain dopaminergic nuclei in mediating the psychomotor consequences of systemic infection.

In healthy mammals, systemic infections typically engender a set of behavioral, psychological, and physiological changes collectively known as “sickness behavior.” Cognitive and affective symptoms include psychomotor retardation, impaired memory, confusion, decreased motivation (e.g., anorexia, adipsia, fatigue), anxiety, and depression (1). Sickness behavior may be conceptualized as an adaptive reorganization of the host's homoeostatic and behavioral priorities to facilitate an immune response, rather than simply a detrimental consequence of infection per se (2).

Animal and human studies suggest that inflammatory cytokines play a pivotal role in mediating sickness-related behaviors by communicating peripheral inflammation to the brain (1,3). The rapid activation of tissue macrophages early in the innate immune response to infection causes the release of cytokines interleukin-6 (IL-6), interleukin-1β (IL-1β), and tumor necrosis factor-α (TNF-α), along with other proinflammatory mediators. Systemic administration of IL-1β or bacterial lipopolysaccharide (LPS) (a potent stimulant of cytokine release) induces sickness symptoms in rodents (1). In humans, cytokine immunotherapy for cancer or hepatitis C frequently produces sickness-related symptoms including psychomotor retardation and depressed mood (3). Similarly, elevated circulating IL-6 levels are associated with depressed mood and memory deficits in healthy volunteers following peripheral injection with bacterial endotoxins (4–6). Peripheral cytokines may access and influence the brain through passive diffusion (at the level of circumventricular organs and choroid plexus), active transport (at the level of brain endothelium), and through the activation of interoceptive afferents, particularly in the vagus nerve, that project to the nucleus of the solitary tract and higher viscerosensory centers (1). Within the brain, there exists a network of cells that produce cytokines and express cytokine receptors (1), where central cytokine production can profoundly influence catecholamine neurotransmission, synaptic plasticity, and neuronal survival (7).

Beyond this evidence, the mechanisms through which peripheral inflammation engenders sickness-related psychomotor and behavioral symptoms remain poorly understood. The current investigation set out to address these issues by assessing the impact of a low-grade peripheral immune stimulus, *Salmonella typhi* polysaccharide, on cognitive performance and brain activity in healthy young men.

## Methods and Materials

### Study Population

Sixteen male student volunteers between 18 and 35 years of age were recruited from University College London. Volunteers were screened by structured interview to ensure that they were healthy, had no previous history of any relevant physical or psychiatric illness, were taking no medication, and were nonsmokers. Volunteers who had received typhoid vaccine in the past 3 years or any other vaccine in the previous 6 months were excluded. Participants were advised not to consume caffeinated beverages or alcohol and to refrain from excessive exercise during the 12 hours prior to testing. They were also advised not to take aspirin, ibuprofen, or antibiotics for 14 days prior to testing. All participants gave their informed consent, and the study was approved by the joint University College London/University College London Hospital Committee on the Ethics of Human Research.

### General Procedure

The study was performed in a double-blind, randomized, crossover-controlled manner. Participants were assessed individually in two separate sessions, a mean of 7 days apart. In session one, a baseline blood sample was drawn and the participant was randomly assigned to one of two experimental conditions (typhoid vaccine or placebo). Injections of *Salmonella typhi* capsular polysaccharide vaccine (.025 mg in .5 mL; Typhim Vi, Aventis Pasteur S.A., Lyon, France) or control saline placebo (.5 mL) were administered intramuscularly into the nondominant deltoid muscle. Typhoid vaccine was selected as a low-grade proinflammatory stimulus, since this vaccine is known to induce increases in circulating proinflammatory cytokine levels with no significant effect on body temperature, a potentially confounding factor (4). To investigate the effect of peripheral inflammation on cognitive and cerebral function, participants performed two tasks during simultaneous functional magnetic resonance brain imaging. The first was a low-grade visual stimulation task and the second was a mentally challenging color-word Stroop task. Tasks were performed 2 hours postinjection, around the time of peak cytokine responses to typhoid vaccine (8). A second blood sample was drawn after scanning (at 3 hours postinjection) for assessment of inflammatory cytokines. Subjective ratings of mood and illness symptoms were taken at baseline, 2 hours, and 3 hours, and body temperature was assessed using a sublingual digital thermometer. The second session was identical to the first except that participants were injected with saline placebo if they had received typhoid vaccine in session one or vice versa.

### Assessment of Mood and Illness Symptoms

Mood and symptoms of illness were assessed with a modified, 36-item version of the Profile of Mood States (POMS) (9), as described previously (4). Six high-loading items were taken from the vigor, tension-anxiety, depression-dejection, and confusion scales of the original POMS, and five items were taken from the fatigue scale. Four extra items were added to assess symptoms associated with mild infection (fever, aching joints, nausea, and headache). Participants were asked to rate how they felt at that moment on a 5-point scale from 0 = not at all to 4 = extremely.

### Low-Grade Visual Stimulation Task

**Figure 1:**
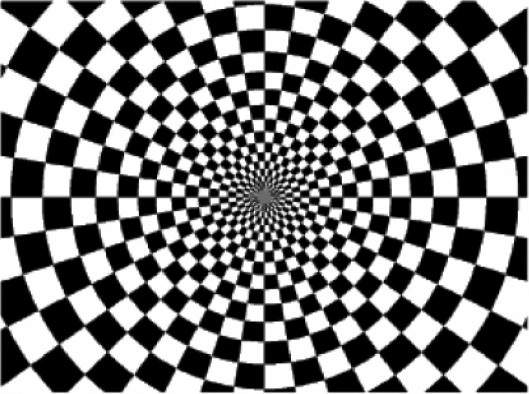
High-contrast flashing checkerboard task used as a potent stimulus of primary visual cortical regions. Participants fixated on a cross at the center of the screen and pressed a key whenever the brightness of the cross changed.

First, to test for a general perturbation of neurovascular coupling during systemic inflammation, each participant underwent a low-level visual stimulation task involving the intermittent presentation of a high-contrast flickering black and white checkerboard stimulus (Figure 1). The stimulus was presented in alternating blocks of 21.6 seconds (on) and 15.1 seconds (off). A total of 20 on blocks and 20 off blocks were presented in two sessions of 6.4 minutes. Participants responded with a right-handed button press to changes in the brightness of a central fixation cross, ensuring attention to the center of the screen. This low-level visual stimulation paradigm is known to induce potent blood oxygenation level-dependent (BOLD) responses in striate and extrastriate visual cortices (V1; Brodmann areas 17 and 18).

### Color-Word Stroop Task

**Figure 2:**
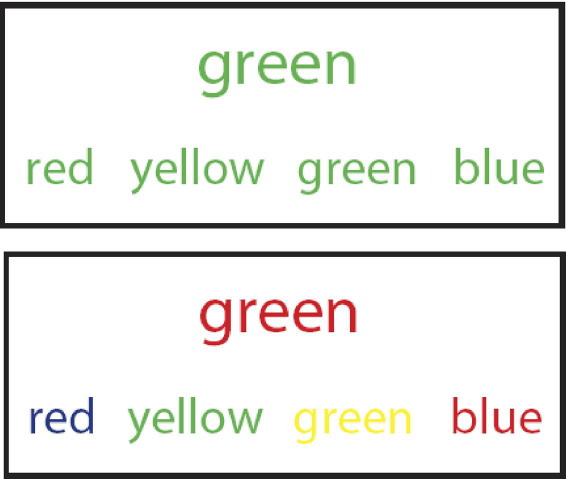
Cognitive color-word Stroop task. Participants pressed a key to select the response word that correctly identified the print color of the target word. Congruent and incongruent conditions were presented separately and examples of these are illustrated here. In the congruent example (upper panel), the correct response is green. In the incongruent example, requiring greater cognitive effort (lower panel), the correct response is red.

Participants performed a second task during fMRI, the color-word Stroop task (Figure 2). This is a very well established task for assessing high-demand cognitive processing, including attentional and executive control processes, via effects of stimulus conflict on psychomotor responses. These functions are known to be compromised by sickness. The task requires the participant to name the ink color of word stimuli, in this case by making a four-choice button press response. The target color word was presented with four possible response words (red, yellow, green, and blue) below. Target words and the order of response words were displayed randomly. Participants were instructed to respond as rapidly as possible to the color of the target word, using a four-button response pad corresponding to the response words below. In the congruent condition, the font color of all words matched both the ink color and semantic meaning of the target word, whereas in the incongruent condition, words were printed in a color incongruent with the color and meaning of the target word. Thus, in incongruent trials, the participant had to overcome distraction from the meaning of the target and ink color of the response choices (Figure 2). Both targets and response words were displayed for 3000 msec, preceded by a central fixation cross presented for 2000 msec. No feedback was given. Trials were presented in five blocks of 36 events with 10,000-msec breaks between blocks. The percentage of incongruent trials varied between blocks and ranged from 14% (two blocks), 28% (one block), to 42% (two blocks), with 17% null events per block.

### Functional Magnetic Resonance Imaging

Functional magnetic resonance imaging (fMRI) was performed using a 1.5T Siemens Sonata magnetic resonance scanner (Siemens, Erlangen, Germany) equipped with a standard head coil. Mild external head restraint was used to minimize head movement during scanning. Visual stimuli were projected onto a screen visible via a mirror on the head coil. Functional brain imaging data were acquired using T2*-weighted echoplanar imaging, sensitive to BOLD contrast. For the visual checkerboard paradigm, the sequence had 24 contiguous slices 2 mm thick, 1 mm interslice gap, no tilt, echo time (TE) 40 msec, and repetition time (TR) 2.16 sec per volume. Three hundred fifty-six volumes were acquired for each participant in two sessions of 6.4 minutes. For the Stroop task, data were acquired with whole-brain coverage using a sequence with 44 contiguous slices, 2 mm slice thickness, 1 mm interslice gap, tilted −30° from intercommissural plane, TE 40 msec, and TR 3.96 sec per volume (10). Two hundred forty-eight volumes were acquired for each participant in a single session of 16.4 minutes.

### Cytokine Analysis

Separate venipunctures were performed at baseline and at 3 hours postvaccination. Blood (10 mL) was drawn into Vacutainer tubes (Becton Dickinson Diagnostics, Oxford, United Kingdom) containing EDTA as anticoagulant, then centrifuged immediately at 1250*g* for 10 minutes at room temperature. Plasma was removed, aliquoted, and frozen at −70°C prior to analysis. Plasma concentrations of IL-6 and TNF-α were assessed using high-sensitivity enzyme-linked immunosorbent assays (ELISAs) from R&D Systems (Oxford, United Kingdom). The limits of detection of these assays were .09 pg/mL (IL-6) and .10 pg/mL (TNF-α), respectively. Plasma interleukin-1 receptor antagonist (IL-1Ra) concentrations were determined by a commercial ELISA from R&D Systems (Oxford, United Kingdom), with a detection limit of 15 pg/mL. All assays had interassay and intra-assay coefficients of variation (CVs) of less than 10%.

### Statistical Analyses

Cytokine levels were analyzed using repeated measures analysis of variance, with treatment (vaccine, placebo) and sample (baseline, postscan) as within-subject factors.

The association between the IL-6 response to vaccine/stress, calculated as the mean increase between baseline and posttask, and mean reaction time (RT) was analyzed using product moment correlations.

Functional MRI datasets were analyzed using SPM5 (Statistical Parametric Mapping [SPM], Wellcome Trust Centre for Neuroimaging, Institute of Neurology, UCL, London, United Kingdom; http://www.fil.ion.ucl.ac.uk/spm). The first five volumes were discarded to allow for T1 equilibration effects. Individual scans were realigned and unwarped, time-corrected, normalized, and spatially smoothed with an 8-mm full width at half maximum (FWHM) Gaussian kernel using SPM methods. A high-pass frequency filter (cutoff 120 sec) and corrections for autocorrelation between scans (AR1) were applied to the time series.

In the visual checkerboard condition, stimulation blocks (checkerboard on; 10 volumes) and rest blocks (checkerboard off; 7 volumes) were modeled as separate regressors. Contrasts of stimulation greater than rest were produced for each subject for both vaccine and placebo conditions and compared within a second level analysis using paired *t* tests. Results for the checkerboard task in primary visual areas are reported at a very liberal threshold of *p* < .01 uncorrected. In analysis of the Stroop task, each event was modeled by a standard synthetic hemodynamic response function at each voxel across the whole brain. Congruent and incongruent trials and errors of commission (response within 3 seconds) and omission (response after 3 seconds) errors were modeled as separate regressors in first-level multiple regression analysis. In the experimental design (see above), null events were included to facilitate identification of differential hemodynamic responses to stochastically ordered stimuli.

The first-level individualized design matrices for each participant were estimated within the general linear model. Effects of task (incongruent and congruent) were computed on a voxelwise basis for each participant for both vaccination and placebo conditions in the form of statistical parametric maps of discrete contrasts. Subsequent second-level paired *t* test analyses were performed on the SPM contrast images for formal inference about population effects. Between-subject effects of mean reaction time and inflammatory response indexed by vaccine-induced change in IL-6 were then determined using regression analysis.

## Results

### Inflammatory Cytokine Response to Vaccination

**Figure 3:**
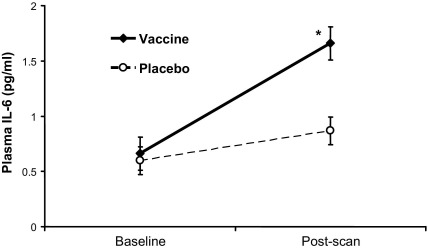
Cytokine response to typhoid vaccination. A high-sensitivity, two-site ELISA was used to measure plasma concentrations of IL-6 in blood samples drawn at the beginning of each session (baseline) and 3 hours following intramuscular injection with either typhoid vaccine or normal saline placebo (postscan). Data are presented as the mean increase in picograms of IL-6 per milliliter of plasma ± SEM in vaccine (solid line) and placebo (dashed line) conditions (*p < .001, *n* = 16; significant treatment by sample interaction, *F* = 7.98, *p =* .013). ELISA, enzyme-linked immunosorbent assay; IL-6, interleukin-6.

Across participants, typhoid vaccination evoked a robust inflammatory response with a greater than twofold increase in plasma IL-6 from .66 ± .38 pmol/L at baseline to 1.66 ± .86 pmol/L at 3 hours (*p* < .001) (Figure 3). The placebo condition evoked a much smaller rise in IL-6 (.60 ± .41 pmol/L at baseline to .87 ± .63 pmol/L at 3 hours, *p =* .05) (Figure 3), consistent with a physiological response to experimental stress (11). There was a significant treatment by sample (baseline and at 3 hours) interaction for IL-6 (*F* = 7.98, *p =* .013). Increases in plasma TNF-α or IL-1Ra did not reach significance, consistent with previous findings (4). There was also no significant effect of vaccination on participants' core body temperature.

### Subjective Response to Vaccination

Vaccination, but not placebo, enhanced subjective ratings of fatigue (*p* < .01) and mental confusion (*p* = .01). There was no effect of vaccine on ratings of illness symptoms, including fever, aching joints, nausea, and headache, and no relationship between cytokine responses and any subjective ratings.

### Peripheral Inflammation and Neural Reactivity During Low-Level Visual Stimulation

**Figure 4:**
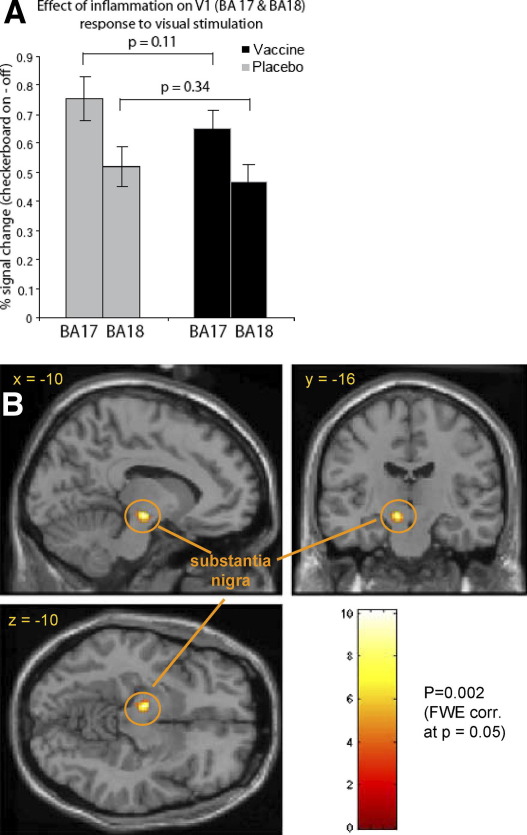
Effect of peripheral inflammation on neural reactivity during low-grade visual stimulation task. **(A)** Mean BOLD signal change (+SEM) during checkerboard stimulus in all voxels probabilistically assigned to Brodmann areas 17 and 18 for vaccine and placebo conditions. There was no significant interaction between mean activation to visual stimulation and inflammatory state in either BA17 (*p =* .11) or BA18 (*p =* .34), confirming no effect of inflammation on neurovascular coupling. **(B)** Interaction between task performance and inflammatory state shows a highly significant change in BOLD signal within the left substantia nigra during performance of flashing checkerboard task (*p =* .002 after whole brain family-wise error [FWE] correction at *p =* .05). BA, Brodmann area; BOLD, blood oxygenation level-dependent; FWE, family-wise error.

As intended, the checkerboard task potently activated early visual cortices in all participants, measured by changes in BOLD signal in V1, reflecting regional vascular reactions coupled to changes in evoked neural activity. Importantly, we observed no differences in the magnitude of the BOLD responses in early visual cortices during inflammation (typhoid vaccine) compared with placebo conditions, indicating no general effect of inflammation on neurovascular coupling (Figure 4A). However, we observed a highly robust (yet unpredicted) inflammation-related change in neural reactivity within the substantia nigra during visual stimulation (Figure 4B). The differential response of the substantia nigra to visual (compared with no visual) stimulation was attenuated in the vaccine condition compared with the placebo condition (Supplement 1). It is noteworthy that this effect was left lateralized, since participants were making right-handed responses during the task.

### Peripheral Inflammation and Reaction Time Performance on Cognitive Stroop Task

**Figure 5:**
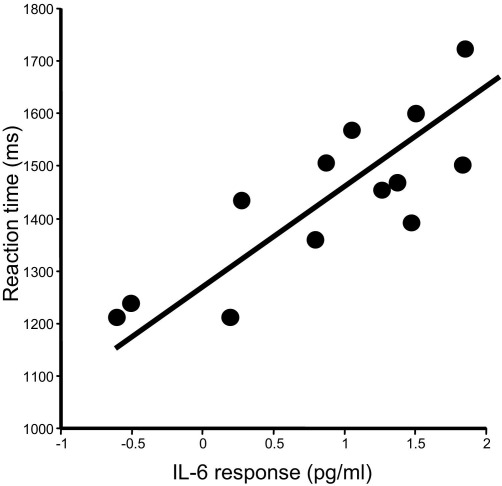
Relationship between IL-6 response to typhoid vaccine and reaction time performance on Stroop task. Scatter plot illustrating the mean key press reaction time in milliseconds (congruent and incongruent conditions grouped) as a function of the mean plasma IL-6 response to typhoid vaccine (increase between baseline and 3 hours) (*r* = .82, *p* < .001). IL-6, interleukin-6.

All participants demonstrated significant increases in response time and number of errors on the incongruent compared with the congruent Stroop task trials [mean RT difference ± SE = 377.6 msec ± 21.6, *t*(23) = 17.46, *p* < .001; mean difference errors ± SE = 4.21 ± .66, *t*(23) = 6.38, *p* < .001]. Vaccination did not affect behavioral measures of Stroop task performance; there were no significant differences in either response time (*p =* 0.31) or number of errors (*p =* 0.79) between typhoid vaccine and placebo conditions. Nevertheless, in the vaccine condition only, the levels of IL-6 (mean increase from baseline) strongly predicted reaction times to both congruent and incongruent Stroop stimuli (vaccine; *r* = .82, *p* < .001, placebo; *p =* .253, ns), indicating that participants with a larger inflammatory (IL-6) response to typhoid vaccine had significantly prolonged reaction time responses (Figure 5). Interestingly, there was no interaction with the degree of stimulus conflict, such that IL-6–related slowing of reaction time for the harder incongruent trials was of the same magnitude as that observed in the easier congruent trials (incongruent trials: *r* = .72, *p =* .006, congruent trials: *r* = .83, *p* < .001). Thus, following vaccination, the level of the inflammatory cytokine IL-6 predicted a general slowing of motor response to stimuli. Moreover, the lack of a superadded effect on responses to incongruent stimuli suggests a precognitive influence of inflammation on response. Due to a paucity of response errors (mean number errors 4 out of 180 trials per session), we were unable to demonstrate a significant relationship between error and IL-6 levels. Reaction time slowing did not correlate significantly with subjective ratings of mood or illness symptoms.

### Relationship Between Neural Reactivity, Reaction Time Performance, and Peripheral Inflammation

**Figure 6:**
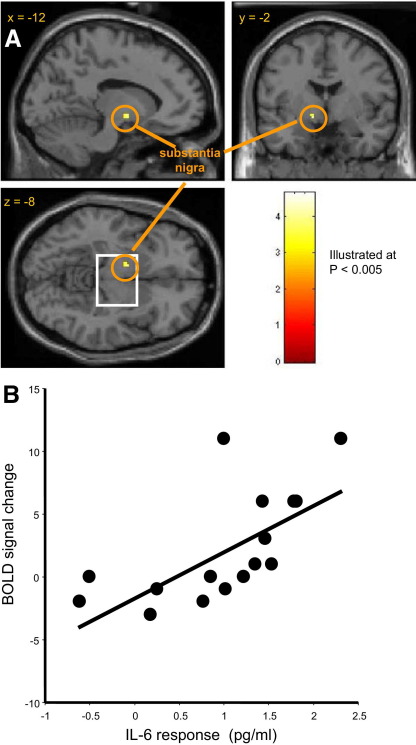
IL-6 response to typhoid vaccine predicts enhanced neural activation within left substantia nigra during Stroop task performance. **(A)** fMRI analysis revealed heightened BOLD activity within left substantia nigra during performance of the cognitive Stroop task in the vaccine condition (threshold *p* < .001, illustrated at *p* < .005 uncorrected). **(B)** Scatter plot illustrating the relationship between substantia nigra activity (BOLD response within an 8 mm region of interest centered on the peak voxel at MNI −12, −2, −8) and mean IL-6 response to typhoid vaccine (*p =* .005). BOLD, blood oxygenation level-dependent; fMRI, functional magnetic resonance imaging; IL-6, interleukin-6; MNI, Montreal Neurological Institute.

We quantified regional brain activity reflecting stimulus processing (both congruent and incongruent stimuli) during the Stroop task in both the vaccine and placebo conditions. We next performed a correlational analysis to determine brain regions that were more activated in individuals with greater IL-6 responses. Participants with a greater IL-6 response to typhoid vaccine demonstrated enhanced neural responses to Stroop stimuli within left substantia nigra (*p* < .001, Figure 6A) (Supplement 2). The correlation of left substantia nigra BOLD response (8 mm region of interest centered on the peak voxel) with IL-6 response to typhoid vaccine (*r* = .67, *p =* .005) is illustrated in Figure 6B. No such relationship was observed in the placebo condition (*p* = .68).

**Figure 7:**
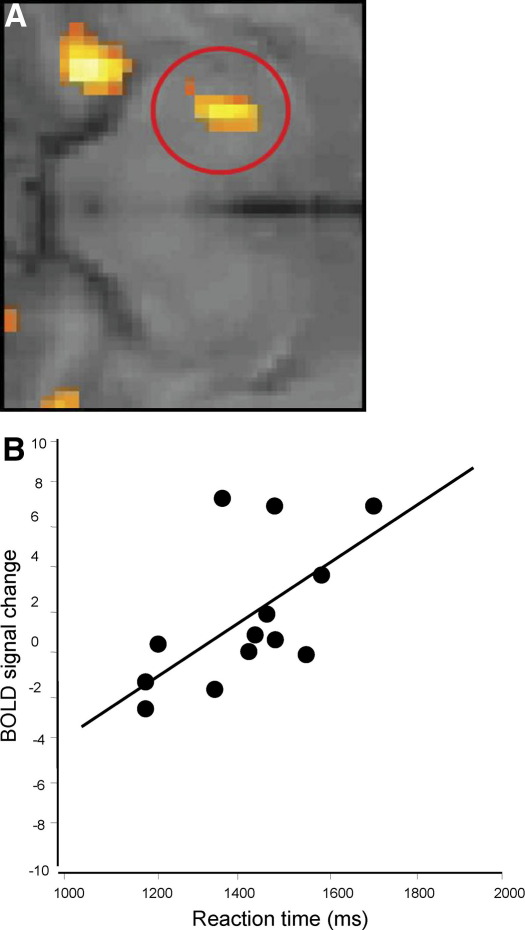
Enhanced substantia nigra activity predicts reaction time slowing. Mean response time on the cognitive Stroop task was significantly correlated with neural activity in the same left substantia nigra region of interest. **(A)** Amplified image of BOLD response in left SN that covaried with reaction time in the vaccine condition. **(B)** Scatter plot illustrating the relationship between substantia nigra activity (BOLD response within an 8 mm region of interest centered on the peak voxel at MNI −12, −2, −8) and mean reaction time in typhoid vaccine condition (*p =* .03). BOLD, blood oxygenation level-dependent; MNI, Montreal Neurological Institute; SN, substantia nigra.

We then performed a similar analysis of the relationship between Stroop task-related activity and individuals' mean response times. Our brain imaging analysis across participants identified a significant correlation between reaction time slowing and BOLD activity changes within the same region of left substantia nigra (Figure 7A and 7B; region-of-interest analysis: *r* = .60, *p =* .03). These findings further implicated the substantia nigra midbrain region in the psychomotor consequences of inflammation. No such relationship was observed in the placebo condition (*p =* .71). Again, due to a paucity of response errors, we were unable to demonstrate a predictive relationship between regional neural (including substantia nigra) activity and performance error on the Stroop task. There was also no correlation between substantia nigra activity and subjective ratings of mood or illness symptoms. It is noteworthy that the location of activity changes within substantia nigra reflecting both IL-6 response and psychomotor slowing was rostral to the more robust effect observed during checkerboard stimulation. This observation suggests a functional topographical organization (behavioral versus perceptual) within the same dopaminergic midbrain nucleus.

## Discussion

This is the first empirical study to directly address the neural mechanisms underlying the psychomotor consequences of peripheral inflammation. Our findings provide objective evidence for a relationship between changes in substantia nigra function and generation of sickness symptoms, notably psychomotor retardation, as a consequence of peripheral inflammation. Substantia nigra activity to performance of a simple motor task (button press) during both low-level visual stimulation and cognitive task performance was modulated by peripheral inflammatory challenge. For the cognitive Stroop task, this effect was most apparent in people with larger inflammatory IL-6 responses to typhoid vaccine, and both IL-6 responses and activity within substantia nigra predicted a precognitive prolongation of motor reaction. The directionality of these effects is noteworthy. During the checkerboard stimulation task, presented and analyzed as a blocked design, inflammation attenuated the normal decrease in BOLD signal within substantia nigra. During the Stroop task, analyzed in an event-related manner (i.e., coupled more directly with motor responses), substantia nigra signal increased with increased reaction time. Although the structure of both tasks is very different, it would appear that the greater BOLD signal reflects a functional perturbation within substantia nigra, the detailed mechanisms of which are presently beyond the resolution of fMRI.

The substantia nigra is a midbrain nucleus, integral to the basal ganglia system, and the major source of dopamine in the brain. The nigrostriatal dopamine pathway is pivotal in the facilitation of movement, signaling of stimulus salience, and driving motivational behavior (12–14). Nigral dopaminergic projection neurons release dopamine within striatal target regions in response to salient stimuli to modulate sensorimotor processing and representations of incentive value across cerebral cortices, thalamus, and amygdala (15). Agonists that potentiate dopaminergic neurotransmission improve the speed of motor responses in animals, whereas striatal dopamine depletion and selective blockade of dopamine D1 or D2 receptors significantly impair performance on reaction time tasks (13,16,17). Similar to our findings, impaired task performance is due to lengthened response latencies rather than deficits in the accuracy of responses, suggestive of a reduction in motivation versus ability to perform (16,17). However, central neural changes related to performance errors have been noted in patients receiving interferon-α (IFN-α) therapy (18). In healthy elderly humans, lower levels of striatal dopamine transporter are associated with slower motor reactions (19), while the loss of nigrostriatal dopaminergic neurons in human Parkinson's disease results in severe deficits in the initiation of motor behavior that can be alleviated with dopaminergic medication (20). Our results extend these observations by implicating the substantia nigra in psychomotor components of sickness behavior mediated by inflammatory cytokines.

Elevated circulating cytokines are associated with psychomotor dysfunction in both animal models and humans. In rodents, systemic administration of bacterial LPS or inflammatory cytokines (notably IL-1β) consistently suppresses locomotor and motivational behaviors, resulting in increased periods of immobility, decreased operant responding for food rewards, and social withdrawal (1). These depressant effects of peripheral IL-1β on behavior are potentiated by IL-6 (21). The motor-suppressing effects of inflammatory challenge are reduced by administration of antibodies against IL-6 (22,23) and attenuated in IL-6 knockout mice (24). Our data suggest that the behavioral effects of peripheral inflammation in humans are mediated by altered dopaminergic neurotransmission. Correspondingly, in rodents, brain dopamine levels are modulated by peripheral administration of IFN-α (a potent inducer of IL-6) and other inflammatory cytokines (25,26). Human patients receiving immunotherapy with IFN-α experience psychomotor slowing and fatigue (3). These symptoms correlate with abnormalities in striatal glucose metabolism, suggesting dysregulation of dopaminergic signaling (27). Elevated circulating IL-6, altered striatal dopaminergic neurotransmission, and psychomotor slowing are also reported in people with clinical depression (28,29). Our novel findings provide direct empirical evidence in humans for involvement of the dopamine system in behavioral consequences of peripheral inflammation, highlighting a role for IL-6 and substantia nigra neural activity in infection-related psychomotor impairments.

Sickness behavior can be considered as the expression of a centrally mediated motivational state that reorganizes the organism's priorities to promote survival (2). Thus, during an infection, psychomotor slowing may minimize energy expenditure and conserve heat, thereby enhancing immune function. Accordingly, cytokine-induced sickness is reflected in a reduction in the number of lever presses that rats will make to obtain a sucrose reward (30). These motivational changes are mirrored in dopamine-depleted rats, which also reduce lever pressing for a preferred food while increasing intake of concurrently available lab chow, thereby reorienting to less effortful food-seeking behaviors (31). Such a failure to sustain behavioral effort in animals that can still initiate normal responding indicates a change in the representation of incentive salience (13). In humans, dopamine antagonists can impair reward-related responses within ventral striatum and diminish behavioral effort to obtain reward (32). Similar motivational deficits occur in patients with Parkinson's disease (13). Our current findings suggest that changes in motivation and incentive salience integral to sickness behavior may, like changes in motor reactivity, arise from inflammation-induced alterations in dopaminergic neuromodulation originating in substantia nigra.

Brain systems controlling behavior may be influenced by peripheral inflammation and cytokines via a set of discrete routes, including activation of vagus nerve or spinal cord interoceptive afferents, passive diffusion of cytokines into cerebrospinal fluid in regions that lack a blood-brain barrier, or active transport across the brain endothelium via cytokine-specific transport molecules (1). Evidence in rodents implicates vagus nerve afferents in the rapid central communication of early inflammatory responses and the modulation of sickness behaviors (33–35). Cytokine receptors are expressed by macrophages and dendritic cells that cluster around vagus nerve perineural sheaths (36) and on vagus nerve sensory afferent terminals such that circulating cytokines can stimulate vagus nerve activity (36). Lipopolysaccharide-mediated induction of peripheral inflammation in rats causes a rapid (within 60 minutes) activation of the primary central projection area of the vagus nerve (nucleus tractus solitarius [NTS]) and subsequent activation of secondary vagus nerve projection areas including parabrachial, paraventricular, and hypothalamic nuclei, amygdala, and bed nucleus of the stria terminalis (33). Vagotomy attenuates this distributed neural response to peripheral inflammatory challenge and blocks corresponding sickness behaviors (34). These data suggest the vagus nerve is important in the early central communication of inflammatory responses observed in the present study.

Animal models demonstrate that chronic peripheral inflammation may also lead to the expression of inflammatory cytokines in microglia within brain stem nuclei including the NTS (33–35). These responses are normally short-lived and tightly regulated by anti-inflammatory cytokines and endogenous glucocorticoids (1). However, in certain neurodegenerative conditions, cytokine-producing microglia may become primed, resulting in exaggerated inflammatory responses that promote neural damage and behavioral deficits (37–40). The substantia nigra contains the greatest density of microglia within the brain and may thus be particularly vulnerable to the consequences of chronic inflammatory responses (41). Microglial activation and cytokine release within substantia nigra precede neural cell loss in rodent models of Parkinson's disease and correlate with indices of disease progression in human patients with Parkinson's disease (42–44). Our own observations of acute inflammation-induced alteration in the functional integrity of the substantia nigra, related to peripheral cytokine response and psychomotor changes, further highlight an intrinsic vulnerability of this region to the effects of inflammation.

Taken together, our findings provide mechanistic insights into the interaction between inflammation and neurocognitive performance, implicating circulating cytokines and midbrain dopaminergic nuclei in mediating the psychomotor consequences of systemic infection. Understanding the mechanisms that link peripheral immune stimuli with neural activity and psychological well-being is fundamental to the development of interventions aimed at controlling the behavioral and depressive symptoms that develop during infection and represent a common comorbidity of inflammatory diseases as diverse as atherosclerosis, obesity, and cancer. Our findings may also be clinically relevant to understanding mechanisms through which systemic inflammation can exacerbate behavioral deficits and disease progression in neurodegenerative disorders such as Parkinson's disease.

## References

[1] Dantzer R., Bluthe R.M., Castanon N., Kelly K.W., Konsman J.-P., Laye S., Ader R. (2007). Cytokines, sickness behavior, and depression. Psychoneuroimmunology.

[2] Hart B.L. (1988). Biological basis of the behavior of sick animals. Neurosci Biobehav Rev.

[3] Capuron L., Miller A.H. (2004). Cytokines and psychopathology: Lessons from interferon-alpha. Biol Psychiatry.

[4] Wright C.E., Strike P.C., Brydon L., Steptoe A. (2005). Acute inflammation and negative mood: Mediation by cytokine activation. Brain Behav Immun.

[5] Krabbe K.S., Reichenberg A., Yirmiya R., Smed A., Pedersen B.K., Bruunsgaard H. (2005). Low-dose endotoxemia and human neuropsychological functions. Brain Behav Immun.

[6] Reichenberg A., Yirmiya R., Schuld A., Kraus T., Haack M., Morag A. (2001). Cytokine-associated emotional and cognitive disturbances in humans. Arch Gen Psychiatry.

[7] Dunn A.J. (2006). Effects of cytokines and infections on brain neurochemistry. Clin Neurosci Res.

[8] Hingorani A.D., Cross J., Kharbanda R.K., Mullen M.J., Bhagat K., Taylor M. (2000). Acute systemic inflammation impairs endothelium-dependent dilatation in humans. Circulation.

[9] McNair D.M., Lorr N., Droppleman L.F. (1981). Manual for the Profile of Mood States.

[10] Deichmann R., Gottfried J.A., Hutton C., Turner R. (2003). Optimized EPI for fMRI studies of the orbitofrontal cortex. Neuroimage.

[11] Brydon L., Edwards S., Mohamed-Ali V., Steptoe A. (2004). Socioeconomic status and stress-induced increases in interleukin-6. Brain Behav Immun.

[12] Groenewegen H.J. (2003). The basal ganglia and motor control. Neural Plast.

[13] Wise R.A. (2004). Dopamine, learning and motivation. Nat Rev Neurosci.

[14] Bunzeck N., Duzel E. (2006). Absolute coding of stimulus novelty in the human substantia nigra/VTA. Neuron.

[15] Horvitz J.C. (2002). Dopamine gating of glutamatergic sensorimotor and incentive motivational input signals to the striatum. Behav Brain Res.

[16] Baunez C., Robbins T.W. (1999). Effects of dopamine depletion of the dorsal striatum and further interaction with subthalamic nucleus lesions in an attentional task in the rat. Neuroscience.

[17] Weed M.R., Gold L.H. (1998). The effects of dopaminergic agents on reaction time in rhesus monkeys. Psychopharmacology (Berl).

[18] Capuron L., Pagnoni G., Demetrashvili M., Woolwine B.J., Nemeroff C.B., Berns G.S. (2005). Anterior cingulate activation and error processing during interferon-alpha treatment. Biol Psychiatry.

[19] van Dyck C.H., Avery R.A., Macavoy M.G., Marek K.L., Quinlan D.M., Baldwin R.M. (2007). Striatal dopamine transporters correlate with simple reaction time in elderly subjects. Neurobiol Aging.

[20] Wichmann T., DeLong M.R. (2003). Functional neuroanatomy of the basal ganglia in Parkinson's disease. Adv Neurol.

[21] Lenczowski M.J., Bluthe R.M., Roth J., Rees G.S., Rushforth D.A., van Dam A.M. (1999). Central administration of rat IL-6 induces HPA activation and fever but not sickness behavior in rats. Am J Physiol.

[22] Pang Y., Fan L.W., Zheng B., Cai Z., Rhodes P.G. (2006). Role of interleukin-6 in lipopolysaccharide-induced brain injury and behavioral dysfunction in neonatal rats. Neuroscience.

[23] Harden L.M., du Plessis I., Poole S., Laburn H.P. (2006). Interleukin-6 and leptin mediate lipopolysaccharide-induced fever and sickness behavior. Physiol Behav.

[24] Bluthe R.M., Michaud B., Poli V., Dantzer R. (2000). Role of IL-6 in cytokine-induced sickness behavior: A study with IL-6 deficient mice. Physiol Behav.

[25] Shuto H., Kataoka Y., Horikawa T., Fujihara N., Oishi R. (1997). Repeated interferon-alpha administration inhibits dopaminergic neural activity in the mouse brain. Brain Res.

[26] Song C., Merali Z., Anisman H. (1999). Variations of nucleus accumbens dopamine and serotonin following systemic interleukin-1, interleukin-2 or interleukin-6 treatment. Neuroscience.

[27] Capuron L., Pagnoni G., Demetrashvili M.F., Lawson D.H., Fornwalt F.B., Woolwine B. (2007). Basal ganglia hypermetabolism and symptoms of fatigue during interferon-alpha therapy. Neuropsychopharmacology.

[28] Maes M., Bosmans E., De Jongh R., Kenis G., Vandoolaeghe E., Neels H. (1997). Increased serum IL-6 and IL-1 receptor antagonist concentrations in major depression and treatment resistant depression. Cytokine.

[29] Martinot M., Bragulat V., Artiges E., Dolle F., Hinnen F., Jouvent R. (2001). Decreased presynaptic dopamine function in the left caudate of depressed patients with affective flattening and psychomotor retardation. Am J Psychiatry.

[30] Merali Z., Brennan K., Brau P., Anisman H. (2003). Dissociating anorexia and anhedonia elicited by interleukin-1beta: Antidepressant and gender effects on responding for “free chow” and “earned” sucrose intake. Psychopharmacology (Berl).

[31] Salamone J.D., Arizzi M.N., Sandoval M.D., Cervone K.M., Aberman J.E. (2002). Dopamine antagonists alter response allocation but do not suppress appetite for food in rats: Contrast between the effects of SKF 83566, raclopride, and fenfluramine on a concurrent choice task. Psychopharmacology (Berl).

[32] Abler B., Erk S., Walter H. (2007). Human reward system activation is modulated by a single dose of olanzapine in healthy subjects in an event-related, double-blind, placebo-controlled fMRI study. Psychopharmacology (Berl).

[33] Konsman J.P., Kelley K., Dantzer R. (1999). Temporal and spatial relationships between lipopolysaccharide-induced expression of Fos, interleukin-1beta and inducible nitric oxide synthase in rat brain. Neuroscience.

[34] Konsman J.P., Luheshi G.N., Bluthe R.M., Dantzer R. (2000). The vagus nerve mediates behavioural depression, but not fever, in response to peripheral immune signals: A functional anatomical analysis. Eur J Neurosci.

[35] Dantzer R., Konsman J.P., Bluthe R.M., Kelley K.W. (2000). Neural and humoral pathways of communication from the immune system to the brain: Parallel or convergent?. Auton Neurosci.

[36] Goehler L.E., Gaykema R.P., Hansen M.K., Anderson K., Maier S.F., Watkins L.R. (2000). Vagal immune-to-brain communication: A visceral chemosensory pathway. Auton Neurosci.

[37] Kim Y.S., Joh T.H. (2006). Microglia, major player in the brain inflammation: Their roles in the pathogenesis of Parkinson's disease. Exp Mol Med.

[38] Allan S.M., Rothwell N.J. (2003). Inflammation in central nervous system injury. Philos Trans R Soc Lond B Biol Sci.

[39] Perry V.H., Cunningham C., Holmes C. (2007). Systemic infections and inflammation affect chronic neurodegeneration. Nat Rev Immunol.

[40] Combrinck M.I., Perry V.H., Cunningham C. (2002). Peripheral infection evokes exaggerated sickness behaviour in pre-clinical murine prion disease. Neuroscience.

[41] Kim W.G., Mohney R.P., Wilson B., Jeohn G.H., Liu B., Hong J.S. (2000). Regional difference in susceptibility to lipopolysaccharide-induced neurotoxicity in the rat brain: Role of microglia. J Neurosci.

[42] Gao H.M., Jiang J., Wilson B., Zhang W., Hong J.S., Liu B. (2002). Microglial activation-mediated delayed and progressive degeneration of rat nigral dopaminergic neurons: Relevance to Parkinson's disease. J Neurochem.

[43] Cicchetti F., Brownell A.L., Williams K., Chen Y.I., Livni E., Isacson O. (2002). Neuroinflammation of the nigrostriatal pathway during progressive 6-OHDA dopamine degeneration in rats monitored by immunohistochemistry and PET imaging. Eur J Neurosci.

[44] Nagatsu T., Sawada M. (2005). Inflammatory process in Parkinson's disease: Role for cytokines. Curr Pharm Des.

