# Supplementary material for: Peripheral Inflammation is Associated with Altered Substantia Nigra Activity and Psychomotor Slowing in Humans
Source: Biol Psychiatry. 2008 Jun 1;63(11):1022–9. doi: 10.1016/j.biopsych.2007.12.007 (PMC2885493; doi:10.1016/j.biopsych.2007.12.007)
Supplement: Supplement 1 [file mmc1.pdf]

Supplement 1

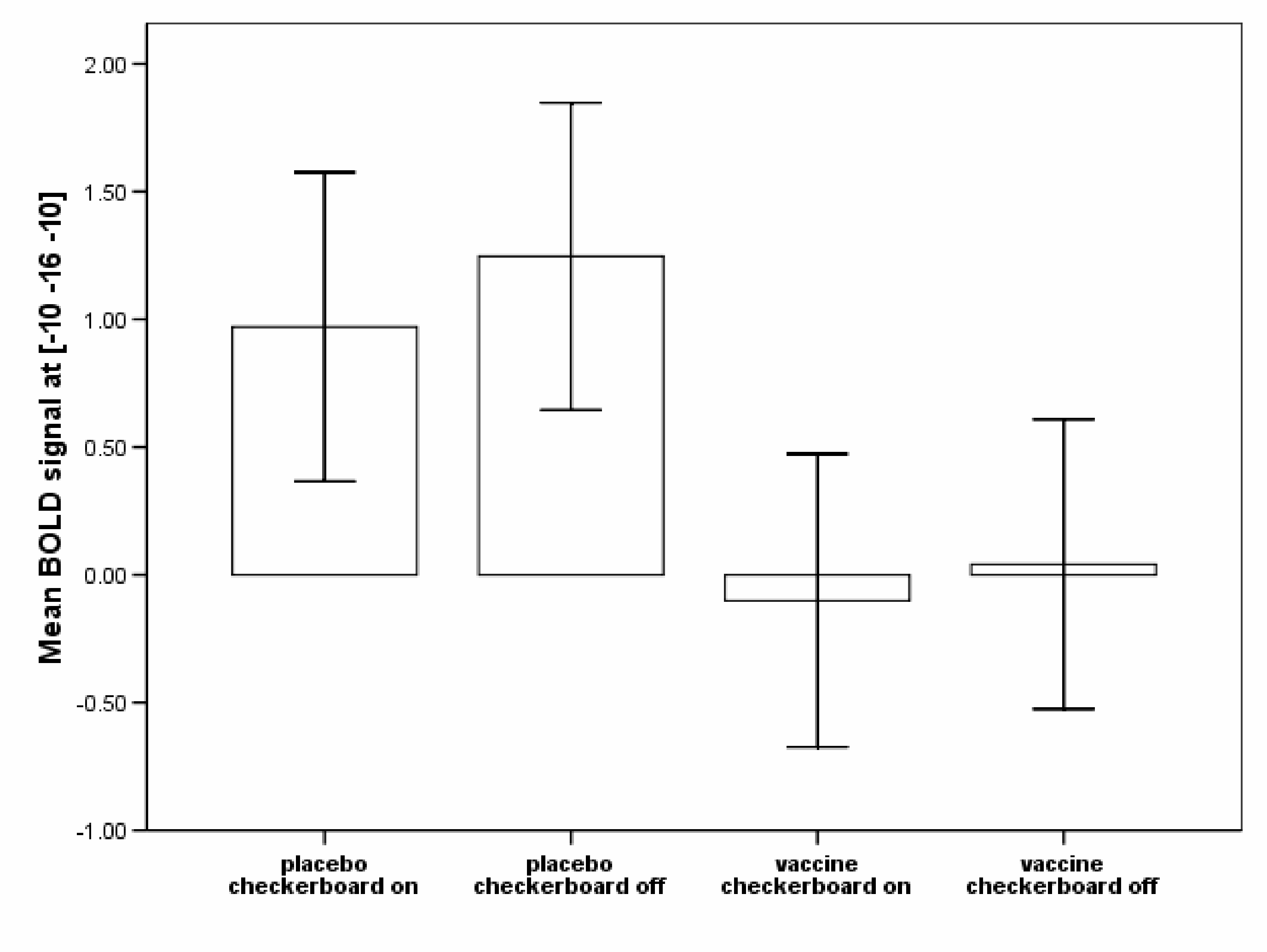

Box-plots illustrating the level of substantia nigra activation, determined by the mean BOLD response at the peak voxel in the left substantia nigra (MNI -10,-16, -10), in vaccine and placebo conditions with checkerboard stimulus on and off.
