# Supplementary material for: Peripheral Inflammation is Associated with Altered Substantia Nigra Activity and Psychomotor Slowing in Humans
Source: Biol Psychiatry. 2008 Jun 1;63(11):1022–9. doi: 10.1016/j.biopsych.2007.12.007 (PMC2885493; doi:10.1016/j.biopsych.2007.12.007)
Supplement: Supplement 2 [file mmc2.doc]

**Supplement 2: Regional brain activity during Stroop task relating to IL-6 response to typhoid vaccine and reaction time performance.**

| Side Region  **Correlation with IL-6** | | MNI  *x y z* | | | *Z score* | *P* |
| --- | --- | --- | --- | --- | --- | --- |
| *L* | *Substantia Nigra* | *-12* | *-2* | *-8* | *3.08* | *P = 0.001* |
| *R* | *Precentral gyrus* | *36* | *-12* | *56* | *2.97* | *P = 0.001* |

**Correlation with RT** *x y z Z score P*

| *R* | *Parahippocampal gyrus* | *24* | *-26* | *-36* | *3.38* | *P < 0.001* |
| --- | --- | --- | --- | --- | --- | --- |
| *R* | *Fusiform gyrus* | 40 | -40 | -32 | 3.22 | *P = 0.001* |
| *R* | *Middle occipital gyrus* | *34* | *-78* | *2* | *3.17* | *P = 0.001* |
| *L* | *Fornix* | -22 | -28 | -8 | 3.11 | *P = 0.001* |
| *L* | *Substantia Nigra* | -14 | -8 | -8 | 2.62 | *P = 0.004* |
